# The Influence of Aging in Solvents on Dental Cements Hardness and Diametral Tensile Strength

**DOI:** 10.3390/ma12152464

**Published:** 2019-08-02

**Authors:** Agata Szczesio-Wlodarczyk, Karolina Rams, Karolina Kopacz, Jerzy Sokolowski, Kinga Bociong

**Affiliations:** 1 University Laboratory of Materials Research, Medical University of Lodz, ul Pomorska 251, 92-213 Lodz, Poland; 2 Department of General Dentistry, Medical University of Lodz, ul, Pomorska 251, 92-213 Lodz, Poland; 3 “DynamoLab” Academic Laboratory of Movement and Human Physical Performance, Medical University of Lodz, ul. Pomorska 251, 92-216 Lodz, Poland

**Keywords:** dental cements, ethanol, water, resistance, DTS, Vickers hardness

## Abstract

Prosthetic materials must exhibit adequate resistance to the oral environment. The aim of this paper was to study the resistance of selected cements used for cementing restorations (Breeze—composite, Adhesor Carbofine—zinc-polycarboxylate and IHDENT–Giz type II—glass-ionomer) against ethanol, soda and green tea solutions. The highest values of hardness and DTS (diametral tensile strength) were obtained by composite cement (HV = 15–31, DTS = 34–45 MPa). Ethanol solution had the greatest impact on the hardness value of composite cement, and soda solution on zinc-polycarboxylate cement. No significant differences were noted in the DTS values of composite cements after immersion in solvents; however, the DTS value of zinc-polycarboxylate cement increased after prolonged immersion time in ethanol and the DTS of glass-ionomer cement (IHDENT Giz type II) clearly decreased after submersion in soda solutions. Variation in pH across the range of 6 (tea) to 9 (soda solution) had a low impact on the properties of dental cements. Extended exposure to solvents appears to worsen the properties of cements.

## 1. Introduction

Dental cements have a wide range of applications in modern dentistry. They are used for luting, fixation and cementation, i.e., luting inlays crowns, bridges, veneers on the prepared tooth. They protect pulp from heat (“thermal insulation”) and from chemical irritation (liners and bases), they also stimulate secondary dentin formation and act as temporary filling material.

The rehabilitation of the stomatognathic system is a very important aspect for patients with partial edentulism. Prosthetic restorations used during treatment often restore lost function of chewing, improve aesthetics and speech [1]. Dental cements constitute an important element during prosthetic treatment. These materials are designed to bond the restoration to the natural teeth of the patient or metallic core. Cements affect the retention of a restoration and protect the exposed dentin against many mechanical, chemical, thermal and bacterial factors. Success of the treatment depends on the proper selection of material used to bond the restoration. As there is currently no ideal cement that can meet all the requirements in terms of mechanical and biological properties, it is important to make an appropriate individual selection for each patient based on the properties of the materials [2,3,4]. 

Nowadays, prosthetic restoration can be performed using composite, zinc-polycarboxylate, glass-ionomer, glass-ionomer reinforced with resin, zinc-phosphate and oxide-zinc-eugenol cements and compomers [5]. Of these, composite cements show the best mechanical properties: Their diametral tensile strength (DTS) ranges from 30 to 60 MPa (zinc phosphate cements approximately 10 MPa, polycarboxylate cements approximately 10 MPa, glass-ionomer cements greater than 15 MPa) and compressive strength from 140 to 200 MPa (zinc phosphate cements approximately 50 MPa, polycarboxylate cements greater than 60 MPa, glass-ionomer cements approximately 100 MPa) [6,7]. These materials consist of an organic matrix and powdered ceramics, e.g., aluminum-boron-bar glass or silanized silica. The filler constitutes from 30% to 80% of the volume of the material, and the size of their particles is from 0.04 to 5.0 µm. The organic phase (matrix) consists of resins such as bisphenol A glycol dimethacrylate (bis-GMA), urethane dimethacrylate (UDMA), hydroxyethyl methacrylate (HEMA), 4-methacryloxyethyl trimellitic anhydride (4-META). Most resin cements contain bis-GMA. Some materials have replaced bis-GMA partially or entirely with bis-EMA resin (ethoxylated bisphenol-A dimethacrylate) or UDMA to reduce their viscosity, eliminating the need for large quantities of diluents such as TEGDMA (triethylene glycol dimethacrylate) and DEGMA (di(ethylene glycol) dimethacrylate) monomers [4,8,9].

Zinc-polycarboxylate cements have been in use for over 30 years and are considered to be the safest cements due to their high biocompatibility. They are available in powder and liquid forms. The powder consists of zinc and magnesium oxide previously subjected to sintering and grinding; it also sometimes contains tin fluoride, which influences the setting time. The liquid form is usually based on a 40% aqueous solution of polyacrylic acid; however, cements can also be made with distilled water, the powder contains oxides particles coated with polyacrylic acid (15–18%). Mixing the powder with the liquid induces formation of complexes called chelates. The reactions occur between metal ions (zinc, calcium) and carboxylic groups derived from polyacrylic acid (liquid) [2,10,11]. 

Zinc-polycarboxylate cements not only offer very favorable biocompatible properties, but they also are characterized by good adhesion to dentine. Unfortunately, such cements also demonstrate poor mechanical properties, such as low compressive strength (67–91 MPa) and high solubility; in addition, due to their plasticity after curing, they are also not viable for cementation of restorations exposed to high short-circuit forces or those with large spans. Zinc-polycarboxylate cements also show low resistance to erosion in an acidic environment, which is a contraindication to use in patients struggling with reflux disease or consuming large amounts of acidic or carbonated drinks [12].

Glass-ionomer cements (GIC) are also available as powders and liquids. The powder consists of calcium-aluminum-fluorosilicate glass. The fluid is an aqueous solution of polyacrylic acid, maleic acid, tartaric acid and itaconic acid [13,14]. The glass-ionomer cements are cured through acid-base reactions comprising a three-step process of dissolving, gelling and hardening. Reactions occur between polyacids (polyanions) and glass (metal cations and fluoride anions) which leads to the formation of a gel matrix. The main advantage of glass ionomer cements is their ability to release fluoride ions, which has a remineralizing effect and prevents the development of caries. They bind physically and chemically with enamel and dentin [13]. Among other properties, they are also distinguished by adequate tensile and compression strength: The compressive strength of glass-ionomer cements is above 130 MPa and this value is sufficient to resist the masticatory forces in the posterior teeth [15]. They also have a similar coefficient of thermal expansion to dentine [15,16]. Glass-ionomer cements are characterized by high sensitivity to both moisture and dehydration. In all binding steps, they have high solubility in water, which can lead to modification of their mechanical properties. However, such materials are also characterized by low initial pH, which can lead to pulp hypersensitivity [3,17]. The above-mentioned cements seem to be most often used in today’s dental prosthetics, hence their resistance to selected solvents are significant. 

Prosthetic cements must be resistant to wide variations in the oral fluid environment, which is influenced by a range of factors including food, drinks and smoking. The aim of the work is to examine the resistance of selected cements used for cementing prosthetic restorations (composite, zinc-polycarboxylate and glass-ionomer) to solvents (75% ethanol, soda solution, green tea) and variations in pH = 5–9.

The following research hypothesis is stated at work: Solvents with different pH (across the range of 6 (tea) to 9 (soda solution)) has an impact on the properties of dental cements.

## 2. Materials and Methods 

Three different cements were selected for the tests: Breeze, Adhesor Carbofine and IHADENTA Giz typ II. Selected cements are a representative example of luting cements (resin, glass-ionomer and zinc-polycarboxylate). Table 1 contains information on tested materials. 

Fifty samples of each cement were made according to the manufacturer’s instructions (Table 1). All samples were shaped as cylinders with a diameter of 6 mm and a height of 3 mm. The material was cured according to manufacturer’s instructions (Table 1) and the samples were placed in a container with distilled water for a period of seven days. 

After seven days, control tests (hardness and DTS) were conducted. Five samples were put in separate containers that contained various substances:

- Solution of baking soda (2.5%);
- Solution of water and ethanol (75%);
- Green tea.

Most foods have a pH close to neutral or acidic. In order to widen the pH range, a 2.5% solution of baking soda was included. Baking soda (sodium bicarbonate) produces OH^−^ ions during hydrolysis, which can accelerate the hydrolysis of dental materials. 

Samples were immersed in the solutions for 1, 7 and 30 days. The pH values of the solutions were measured using litmus paper and a pH meter (CPI-505, Elmetron, Zabrze, Polska). The obtained results are presented in Table 2.

The hardness of Breeze and Adhesor Carbofine cements were measured using the Vickers method. The Zwick ZHV2-m hardness tester (Zwick-Roell, Ulm, Germany) was used for the tests. The applied load was 1000 g and the penetration time was 10 s. Eleven measurements were performed on three out of five samples from container for each material at specific time intervals (1, 7 and 30 days).

For testing the diametral tensile strength (DTS), a Zwick Roell Z020 universal strength machine (Germany) was used. The traverse speed was 2 mm/min. The measurement was made on five samples from each material at specific time intervals (1, 7 and 30 days) making a total of 15 samples. The DTS values were calculated using the Formula (1):

(1) $$DTS=\frac{2F}{\pi dh}(\mathrm{MPa})$$

*F*—Compressive force, which caused the destruction of the sample (N)

*d*—Diameter of the sample (mm)

*h*—Height of the sample (mm).

For statistical calculations, Statistica v. 13.1 (Statsoft, Kraków, Poland) was used. The normality of the distribution of data was confirmed using the Shapiro—Wilk test; depending on the result, either parametric (F-test) or non-parametric (Kruskal—Wallis test) tests were used for statistical analysis (alpha = 0.05). Equality of variance was tested with Levene’s test.

## 3. Results and Discussion 

**Figure 1:**
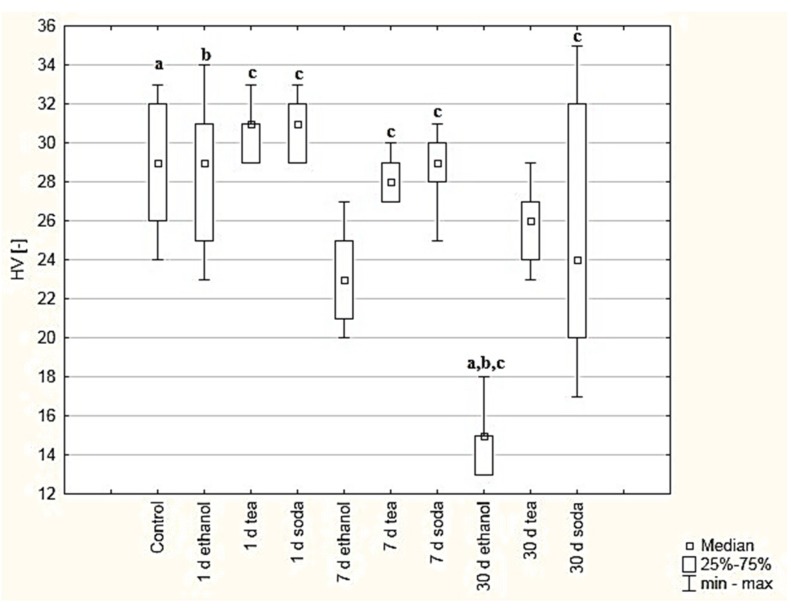
Box and whiskers plot of Vickers hardness of samples. Values were obtained for samples made of Breeze cement treated with ethanol (75%), green tea and baking soda (2.5%) solutions after one, seven or 30 days and samples immersed for seven days in distilled water as control. Statistically significant differences were detected between: (a) Control group vs. 30 days in ethanol solution, (b) 1 day in ethanol vs. 30 days in ethanol solution, (c) 30 days in ethanol solution vs. 1 day in tea solution, 7 day in tea solution, 1 day in soda solution, 7 day in soda solution, 30 days in soda solution.

**Figure 2:**
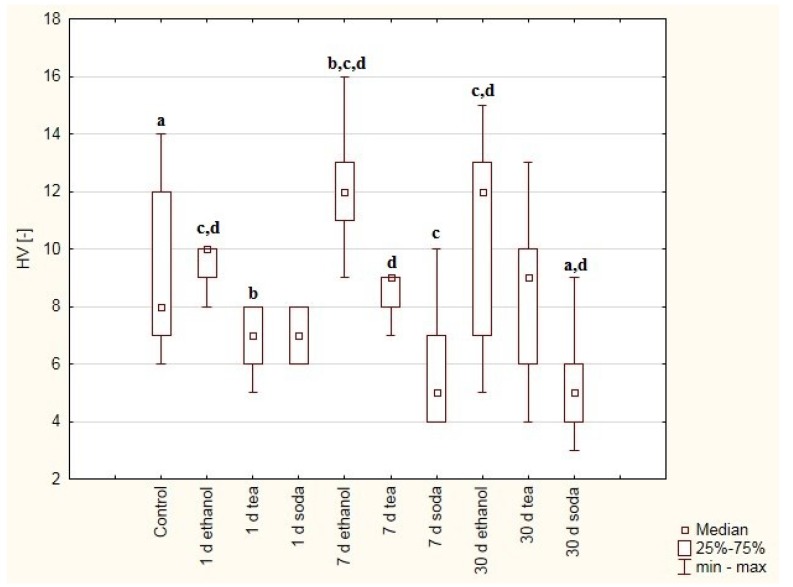
Box and whiskers plot of Vickers hardness. Values were obtained for samples made of Adhesor Carbonfine cement treated with ethanol (75%), green tea and baking soda (2.5%) solutions after one, seven or 30 days and samples immersed for seven days in distilled water as control. Statistically significant differences were detected between: (a) Control group vs. 30 days in soda solution, (b) 1 day in tea solution vs. 7 days in ethanol solution, (c) 7 days in soda solution vs. 1 day in ethanol solution, 7 days in ethanol solution, 30 days in ethanol solution, (d) 30 days in soda solution vs. 1 day in ethanol solution, 7 days in ethanol solution, 30 days in ethanol solution, 7 days in tea solution.

**Figure 3:**
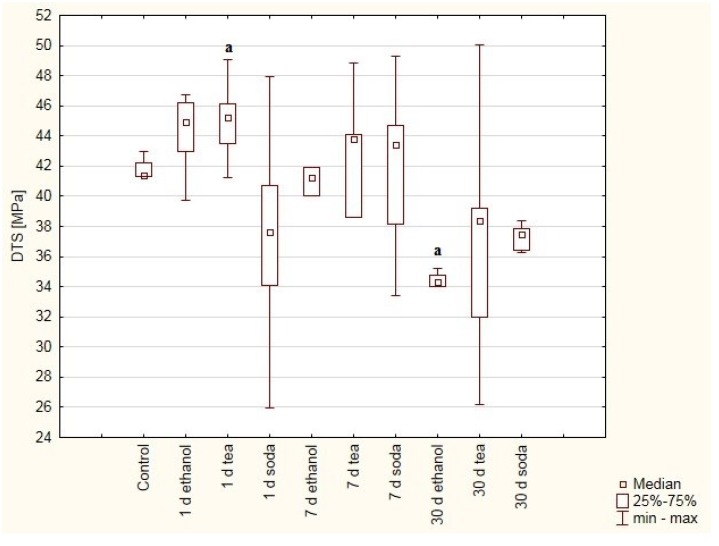
Box and whiskers plot of diametral tensile strength (DTS). Values were obtained for samples made of Breeze cement treated with ethanol (75%), green tea and baking soda (2.5%) solutions after one, seven or 30 days and samples immersed for seven days in distilled water as control. Statistically significant differences were detected between: (a) 1 day in tea solution vs. 30 days in ethanol solution.

**Figure 4:**
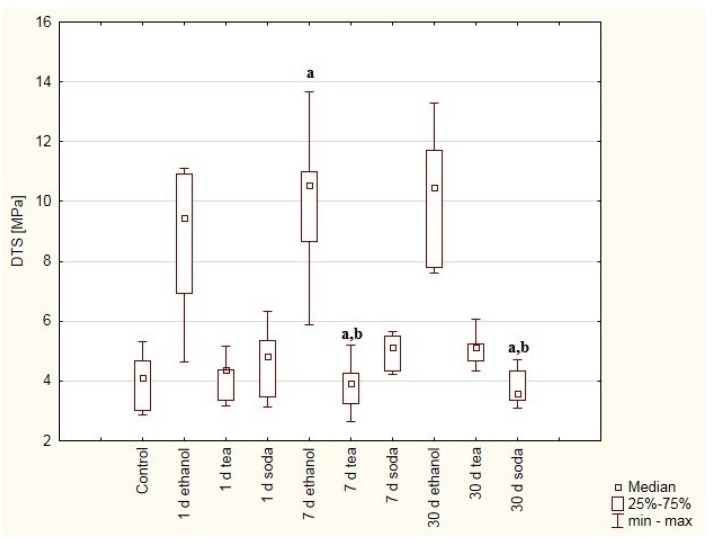
Box and whiskers plot of diametral tensile strength (DTS). Values were obtained for samples made of Adhesor Carbonfine cement treated with ethanol (75%), green tea and baking soda (2.5%) solutions after one, seven or 30 days and samples immersed for seven days in distilled water as control. Statistically significant differences were detected between: (a) 7 days in ethanol solution vs. 30 days in soda solution, 7 days in tea solution, (b) 30 days in tea solution vs. 30 days in soda solution, 7 days in tea solution.

**Figure 5:**
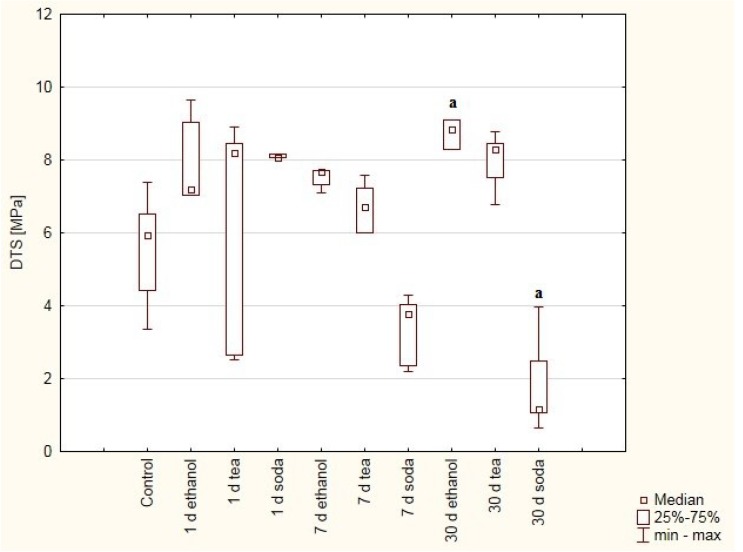
Box and whiskers plot of diametral tensile strength (DTS). Values were obtained for samples made of IHDENT Giz typ II cement treated with ethanol (75%), green tea and baking soda (2.5%) solutions after one, seven or 30 days and samples immersed for seven days in distilled water as control. Significant statistical differences were detected between: (a) 30 days in ethanol solution vs. 30 days in soda solution.

The composite cements, *viz.* Breeze and Adhesor Carbofine, were found to have a higher hardness than the zinc-polycarboxylate cement (Figure 1 and Figure 2). DTS results indicate that Breeze has the highest strength (Figure 3, Figure 4 and Figure 5). Cements modificated with resin matrix demonstrate better properties than traditional cements and tend to be more popular [18]. The research hypothesis was not rejected. But it should be emphaticized that immersion in solutions of pH 6 (tea)–9 (soda solution) has little impact on the properties of the dental cements. The impact depends on the material and solution composition.

The hardness of the composite cement (Breeze) (Figure 1) stored in ethanol solution was found to decrease with time. This tendency is also seen for soda and tea solutions, but no statistically significant difference was found between the two. Significant differences were found between:

- Control group vs. 30 days in ethanol solution (*p* = 0.0006);
- 1 day in ethanol vs. 30 days in ethanol solution (*p* = 0.0008);
- 30 days in ethanol solution vs. 1 day in tea solution (*p* = 0.0000), 7 day in tea solution (*p* = 0.0017), 1 day in soda solution (*p* = 0.0000), 7 day in soda solution (*p* = 0.0006), 30 days in soda solution (*p* = 0.0459).

Although no studies have so far examined the effect of aging in various solvents on the properties of resin cements, Breeze has a similar structure and composition to that of a dental composite with a resin matrix. In contrast to conventional dental resin, cements are most often characterized by the addition of a chemical catalyst, which allows for the dual polymerization of the material. In addition, fluoride can be added as an anticaries agent, and is used to be competitive with GICs. To simplify the cementation procedure, self-adhesive resin cements consisting of adhesive monomers were designed [19]. The fracture toughness values of various dental composites are known to decrease after aging in ethanol solution for six months [20]. Ageing of dental composites in ethanol solution resulted in the elution of residual, unreacted monomers, filler/matrix interfacial failure, and reduction of the mechanical properties [20,21,22,23,24,25,26,27,28]. This can be explained by the structure and properties of the resin matrix. Some dental resins, such as Bis-GMA and HEMA, absorb a significant proportion of ethanol and water molecules thanks to their hydrophilic properties. Ethanol is considered to be good solvent of dental composites: Ethanol and the dimethacrylate resins used in these materials have similar Hoy’s solubility parameters (26.1 and 19.2–23.6 (J/cm^3^)^½^ respectively) [29,30]. Hence, when immersed in ethanol, Breeze swells, resulting in lower moduli of elasticity and strength [31]. 

The hardness of zinc-polycarboxylate cement (Adhesor Carbofine) was found to decrease with duration of immersion in soda solution (Figure 2). No similar relationship was noticed for the soda or tea solutions.

Statistical analysis of hardness results allowed to show significant statistical differences between:

- Control group vs. 30 days in soda solution (*p* = 0.0163),
- 1 day in tea solution vs. 7 days in ethanol solution (*p* = 0.0021),
- 7 days in soda solution vs. 1 day in ethanol solution (*p* = 0.0167), 7 days in ethanol solution (*p* = 0.0000), 30 days in ethanol solution (*p* = 0.0108),
- 30 days in soda solution vs. 1 day in ethanol solution (*p* = 0.0022), 7 days in ethanol solution (*p* = 0.0000), 30 days in ethanol solution (*p* = 0.0013), 7 day in tea solution (*p* = 0.0336).

Adhesor Cabofine belongs to the group of zinc-polycarboxylate cements whose curing reaction consists of the formation of a complex binding between metal ions (zinc, calcium) and carboxylic groups derived from polyacrylic acid. The hardness of zinc-polycarboxylate cements has been found to increase after 35 days in distilled water [32]. This has been attributed to the presence of a solid polycarboxylic phase around the oxides (e.g., zinc oxide) responsible for curing. In addition, glass-ionomer cements and zinc-polycarboxylate cements have been found to be the most soluble of various tested cements [33]. As a result of solubility and sorption, margin integrity tends to reduce, resulting in improved surface properties and aesthetics. Water sorption adversely affects bending strength and hardness [33]. 

Dental cements were found demonstrate greater solubility with duration of immersion in the test solutions, at all tested pH values (pH 3, 7 and 9) [34]. However, for the Adhesor Carbofine cement samples in the present study, no great differences in solubility were observed for samples treated with tea and ethanol solution. Interestingly, a clear downward trend in the value of HV hardness was observed when the cement was immersed in soda solution. These findings can be explained by the fact that the cement contains zinc, magnesium and aluminum oxides, which are higher in the electrochemical series than sodium. Sodium, as a more active element, displaces the less active metal from the salt compound.

Few studies examine the strength properties of prosthetic cements; nevertheless, the most common methods used are the three-point bending and compression tests. The three-point bending test is very important to assess the suitability of materials. It is recognized by the International Organization for Standardization (ISO) as a valid strength test of composite materials [35]. However, it should be noted that sample preparation is difficult and may result in heterogeneous results being obtained for the degree of polymerization. Additionally, real dental restorations are several times smaller and this method is “clinically” unfounded. Furthermore, the tensile strength values of dental materials have greater clinical value than compressive strength, because many clinical failures are due to tensile forces [36]. Composite cements have been found to demonstrate higher DTS values (44 MPa) than zinc phosphate cements [37], with similar results being obtained in other studies (e.g., Li and White [6]., Kim et al. [38]). Sokolowski et al. [7] obtained similar result for a composite cement, i.e., Breeze.

Although it is difficult to compare our findings with those of previous studies, assuming that resin cements consist of similar components as dental composites, it would be reasonable to assume that the two sets of materials have similar strength properties. The DTS values for composite materials for fillings are usually in the range of 30–55 MPa [39,40,41]. Although some variation was observed between the mean DTS values of the Breeze cement, it is within the limits of measurement error (Figure 3). Significant differences in hardness were found between:

- 1 day in tea solution vs. 30 days in ethanol solution (*p* = 0.0321).

The mean DTS value of Adhesor Carbofine cement increased with duration of immersion in ethanol solution (Figure 4). The DTS values of the samples immersed in the tea and soda solutions are very similar and no differences were found. Significant differences in hardness were found between:

- 7 days in ethanol solution vs. 30 days in soda solution (*p* = 0.0439), 7 days in tea solution (0.0439).
- 30 days in tea solution vs. 30 days in soda solution (*p* = 0.0348), 7 days in tea solution (0.0348).

No information is available in the literature regarding the DTS of zinc-polycarboxylic cement (Adhesor Carbofine). However, literature findings indicate that the compressive strength of this material and of other tested zinc-polycarboxylate cements increased after 30 days in water. The reaction between the metal ions with the polyacrylic acid reaches stabilization after one day; therefore, some changes of the mechanical properties can be deduced after this time. This observation may be explained by the presence of the unreacted phase (solid polycarboxylate phase around the zinc oxide or other metal oxides), which can further react, causing hardening over time [32]. Our findings indicate that the DTS values of the samples contained in the ethanol solution increase according to time of immersion. The setting time of cement is believed to be stabilized and extended by the addition of alcohol to the fluid of zinc-polycarboxylic cements.

The highest DTS values of glass ionomer cement (IHDENT Giz type II) were observed for samples treated with ethanol solution after seven or 30 days. The lowest recorded values were for samples immersed in soda solution (Figure 5). Significant differences in hardness were found between:

- 30 days in ethanol solution vs. 30 days in soda solution (*p* = 0.0093).

**Figure 6:**
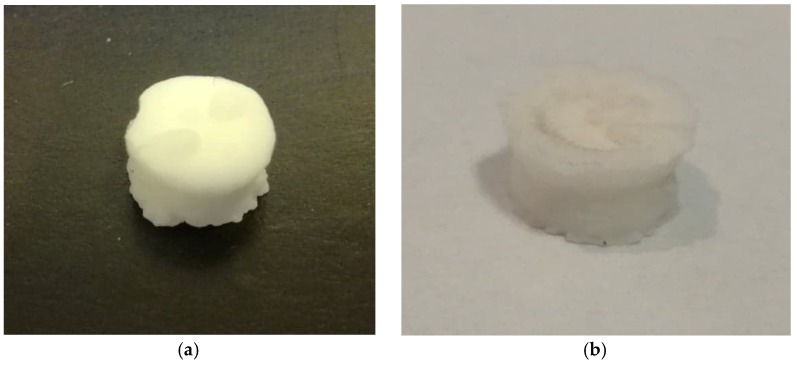
Samples made of IHDENT Giz type II ionomer glass cement removed from the soda solution after (**a**) 7 days and (**b**) 30 days.

It should be emphasized that the mechanical strength of glass ionomer cements depends on the water balance during the curing process. This process is based on acid-base reaction in which an acid reacts with the salts present in the powder; the process causes the release of metal ions and the formation and precipitation of polyolefin salts [42]. The initial curing is followed by a maturation process, which takes place more slowly. It was observed to be disturbed by the presence of soda solution [43]. As with the Adhesor Carbofine cement, the presence of sodium can cause changes in ionic reactions by displacing less active metals from the salt compound. Large changes were observed in the appearance of samples, which delaminated and crumbled after exposure to soda (Figure 6). The DTS results for this material are characterized by low values, in other studies values for GIC are higher (>20 MPa), however, in these studies GIC for temporary fillings was tested [44]. It is know that the highest strength were obtained by the restorative glass-ionomers in comparison to luting type [45].

## 4. Conclusions

1. The immersion in solvents (tea, ethanol and baking soda solutions) have influence on the diametral tensile strength and the hardness of analyzed cements. The impact depends on the material and solution composition.

2. Of the tested cements, composite cement obtained the highest hardness and diametral tensile strength values.

3. Ethanol affects studied composite cement and causes its diametral tensile strength and hardness to deteriorate in time. 

4. Baking soda solution affects the properties of zinc-polycarboxylate. The prolongation of aging time results in a significant reduction in the hardness of the cement. 

5. Baking soda solution influences glass-ionomer properties. Glass-ionomer samples immersed in a baking soda solution after seven and 30 days showed changes in appearance and DTS value.

## Figures and Tables

**Table 1:** The composition of tested materials.

Manufacturer	Material	Type	Composition	Preparation and Curing
Jeneric Pentron (Orange, CA, USA)	Breeze	Self-adhesive resin cement	Bisphenol A glycol dimethacrylate (bis-GMA), urethane dimethacrylate (UDMA), triethylene glycol dimethacrylate (TEGDMA), hydroxyethyl methacrylate (HEMA), 4-methacryloxyethyl trimellitic anhydride (4-MET), silanized barium glass, silica, BiOCl, curing system	Mixing: Self-mixing syringe, curing: Polymerized using a 3M ESPE EliparTM S10 diode lamp on the top and bottom surface of the sample as recommended by the manufacturer for 20 s
SpofaDental (Jičín, Czech Republic)	Adhesor Carbofine	Zinc-polycarboxylate cement	Powder: Zinc oxide, magnesium oxide, aluminum oxide, boric acid, liquid: Acrylic acid, maleic anhydride, distilled water	Mixing: 1:1 (power:liquid) mixing ratio recommended by manufacturer; curing: 7 min self-curing in plastic zip bag
Ihde Dental AG (Gommiswald, Switzerland)	Glass-ionomer cement (Ihdent^®^ GIZ^®^ fil Typ II)	Glass ionomer cement	Aluminum-fluoride-silicate powder, iron oxide, polyacrylic acid	Mixing: 1.8–2.2 g of powder per 1 g of liquid (mixing ratio recommended by manufacturer); curing: 5–8 min self-curing in plastic zip bag

**Table 2:** The results obtained during the pH measurement of the solutions.

Solution	pH (Litmus Paper)	pH (pH-Meter)
Green tea	5–6	6
Ethanol:water (75%)	7	8
Soda solution (2.5%)	9	9
